# Concerned Whether You’ll Make It in Life? Status Anxiety Uniquely Explains Job Satisfaction

**DOI:** 10.3389/fpsyg.2020.01523

**Published:** 2020-07-10

**Authors:** Anna Keshabyan, Martin V. Day

**Affiliations:** Department of Psychology, Memorial University of Newfoundland, St. John’s, NL, Canada

**Keywords:** status anxiety, job satisfaction, job insecurity, occupational self-efficacy, distributive justice, procedural justice, interactional justice, well-being

## Abstract

Ever feel concerned that you may not achieve your career goals or feel worried about where your life is going? Such examples may reflect the experience of status anxiety, that is, concerns that one may be stuck or not able to move up in life, or worries that one may be too low in standing compared to society’s standards. Status anxiety is believed to be exacerbated by economic inequality and negatively affect well-being. While job satisfaction is an important determinant of well-being, no research has examined whether status anxiety can also help explain people’s satisfaction with their jobs. We tested whether status anxiety differs from other organizational constructs and uniquely relates to job satisfaction among full-time working adults. In a pilot study, we found that status anxiety is separate from the concept of job insecurity (e.g., perceived threat of job loss). Results of our main study also indicated that higher status anxiety significantly predicted lower job satisfaction beyond several other indicators of organizational attitudes (job insecurity, occupational self-efficacy, distributive, procedural, and interactional justice), as well as the tendency to seek status and several background factors (e.g., income, education, perceived socioeconomic status). We discuss the unique role of status anxiety in job satisfaction and the implications of this research to our understanding of status concerns, as well as organizational attitudes and policies.

## Introduction

Many people aspire to advance their position in society. There are a variety of advantages to having higher status, including greater influence and perceived competence, as well as higher quality of life and well-being (Marmot, 2003; Conger and Donnellan, 2007; Li et al., 2016; Oh et al., 2020). The desire and prospect of higher socioeconomic status can motivate many important outcomes, such as educational and career achievement (Haller and Portes, 1973; Zukin and Maguire, 2004; Shane et al., 2012; Gerbasi and Prentice, 2013; Shane and Heckhausen, 2016; Browman et al., 2017; Elliot and Hulleman, 2017). However, there is almost always a higher status that can be attained, and the vast majority never reach the very top. Thus, status ambition can also have downsides.

Notably, the desire for higher status may be accompanied by the experience of status anxiety. As first broadly conceptualized by de Botton (2004), status anxiety involves concerns that one does not fulfill society’s definition of success. It can be reflected in worries about not moving up, being too low or stuck in a current position in society, or becoming lower in status (de Botton, 2004; see also Jensen, 2006; Gill, 2015). Status anxiety is not trivial. Such status-related concerns are believed to be a contributing factor in chronic stress, which can negatively affect multiple aspects of people’s lives, including physical and mental health and overall well-being (Wilkinson and Pickett, 2009). The experience of status anxiety is also believed to play an important role in explaining how higher economic inequality is associated with lower well-being outcomes (Wilkinson and Pickett, 2009; Pickett and Wilkinson, 2015; Buttrick et al., 2017; Paskov et al., 2017; Layte et al., 2019). Moreover, emerging research has linked various indicators of status anxiety with lower subjective well-being (Delhey and Dragolov, 2014; Layte and Whelan, 2014; Day and Fiske, manuscript in preparation).

Although there are many aspects of well-being, one critical component for working adults is their satisfaction with their job (Henne and Locke, 1985; Judge and Watanabe, 1993). Job satisfaction typically reflects a global sense of contentment and need fulfillment a person gets from his/her job and its conditions, including daily tasks, salary, relationships with colleagues and management, and overall organizational culture and procedures (Locke, 1976; Spector, 1997). Research indicates that higher job satisfaction is positively related to perceived life meaningfulness and positive mental health (Faragher et al., 2005; Slemp and Vella-Brodrick, 2014; Allan et al., 2018) and negatively related to depression and overall stress (Batlis, 1980; Judge et al., 2002; Allan et al., 2018). Job satisfaction appears to be consequential in other ways. While the associations tend to be moderate or weak, higher job satisfaction is related to better job and organizational performance (Judge et al., 2001; Bakotić, 2016), as well as lower absenteeism and employee turnover (Carsten and Spector, 1987; Johns, 1997; Medina, 2012).

Although job satisfaction has been associated with a variety of factors, much of this research has focused on job and workplace-related variables (Hackman and Oldham, 1976; Spector, 1997; Syptak et al., 1999). Despite the importance of job satisfaction, the role of status ambition in workplace achievement, and the potentially elucidating role of status anxiety in well-being, there appears to be scarce research into whether the experience of status anxiety may help explain job satisfaction (Judge et al., 2017). Thus, the present research seeks to address this issue and broaden the existing literature by providing a first test of the possible relationship between status anxiety and job satisfaction. In particular, we examine whether status anxiety differs from other organizational constructs and uniquely relates to lower job satisfaction.

Next, we explain why status anxiety may contribute to lower job satisfaction. After, we review some relevant predictors of job satisfaction (e.g., job insecurity, organizational justice beliefs) and explain our present research approach.

### Status Anxiety and Job Satisfaction

Status anxiety is not a work- or job-specific evaluation. It involves general concerns relating to one’s position in society (de Botton, 2004; Wilkinson and Pickett, 2009; Gill, 2015). However, there are several reasons to believe that it may affect job satisfaction. As discussed above, status anxiety is believed to affect mental health and has been found to relate to indicators of well-being. As job satisfaction can be a component of well-being for working-age adults, it seems plausible that the experience of status anxiety may also relate to it.

The general pursuit of status and its relation to the workplace provides some insight into this possible association. One’s occupation can be a major component of a person’s perceived status (e.g., occupational prestige, salary, societal value; Haller and Portes, 1973), but not all workers attain their desired workplace rank or advance at the same pace as their expectations (Rindfuss et al., 1999). The associated experience of status anxiety among some individuals may thus reflect a lack of fulfillment (e.g., with societal rank), which if extended to workplace fulfillment may contribute to job dissatisfaction (Locke, 1976). Moreover, concerns and worries about achieving status can generally be unpleasant (de Botton, 2004; Wilkinson and Pickett, 2009); thus, the experience of status anxiety may negatively affect the overall enjoyment of one’s work. Such ruminations about one’s status are not believed to motivate action, but rather may be cognitively taxing or distracting, which may interfere with work tasks and thus job satisfaction.

Moreover, as status anxiety may be heightened by a scarcity of advancement opportunities (e.g., concerns about being trapped at certain level, not moving up) or lowering of one’s status, it may also lead to lower job satisfaction to the extent that such status-related situations (e.g., insufficient promotions, possibility of demotion, or precarious employment; Kalleberg, 2009, 2018) are salient or make people uncertain about their commitment to their organization (Mottaz, 1988). In addition, status anxiety may lower job satisfaction through weakening workplace relationships. As people appear to place greater weight on upward comparisons (Boyce et al., 2010; Kim et al., 2018), the success of others in the workplace may heighten status anxiety, as well as malicious feelings of envy toward them (Day and Fiske, manuscript in preparation). This may negatively affect the quality of social bonds in the workplace and overall attitudes toward one’s job.

Based on the general reasoning and insights discussed, we hypothesize that there will be a negative relationship between status anxiety and job satisfaction. In particular, we expect this association to exist beyond other notable predictors of job satisfaction reviewed below.

### Additional Predictors of Job Satisfaction

Job satisfaction is related to a multitude of organizational attitudes. Through a review of the literature, we have identified several applicable factors. This includes job insecurity, occupational self-efficacy, and perceptions of distributive, procedural, and interactional justice (McFarlin and Sweeney, 1992; Cohen-Charash and Spector, 2001; Ladebo et al., 2008; Borgogni et al., 2010; Bernhard-Oettel et al., 2011). Thus, in the present research, we will examine whether status anxiety associates with job satisfaction beyond these other organizational attitudes, with a particular focus on distinguishing the role of status anxiety from job insecurity. We believe that status anxiety is a separate construct from job insecurity, but we acknowledge the potential overlap. Given that a validated measure of status anxiety has only recently been examined (Day and Fiske, manuscript in preparation), we take this opportunity to further examine the validity of this construct.

#### Job Insecurity

Job insecurity refers to the worry a person feels about the future stability of their employment situation. Key elements include the affective reaction to the subjective threat of job loss or diminished working conditions (Shoss, 2017; Jiang and Lavaysse, 2018). Job insecurity is associated with a variety of outcomes including poorer quality of life, as well as lower self-esteem, self-efficacy, and, notably, job satisfaction (Heaney et al., 1994; De Witte, 1999; Ferrie, 2001; Sverke et al., 2002; Ito and Brotheridge, 2007; Reisel et al., 2010; Bernhard-Oettel et al., 2011; Debus et al., 2014; Keim et al., 2014). Job insecurity and status anxiety both appear to involve concerns with descending to a lower social position. However, there are a number of important distinctions between these concepts. Job insecurity is centered on the fear of losing one’s job or its conditions. Status anxiety involves concerns about one’s status in general, which may or may not include one’s employment status. Moreover, status anxiety also involves concerns about not being able to advance to a higher status, being stuck at a status level, or currently being too low in status. Job insecurity generally does not involve broader concerns about advancement or societal rank.

Despite these conceptual differences, in the present research, we will conduct two tests to further increase confidence in the distinctness of job insecurity and status anxiety (e.g., Clark and Watson, 1995). This includes the possible unique association between status anxiety and job satisfaction. As we explain later, this will be done through confirmatory factor analyses in a pilot study and multiple regression analyses in our main study.

#### Occupational Self-Efficacy

Another relevant factor, occupational self-efficacy, stands for the feeling that one has adequate skills and abilities to complete job-related tasks and solve problems and has perceived competence in terms of work-relevant subject matter (Borgogni et al., 2010). Employees who are confident in their ability to perform at work are typically more satisfied with their jobs and organizations (Judge and Bono, 2001; Borgogni et al., 2010; Gabriel et al., 2014; Maggiori et al., 2016; Guarnaccia et al., 2018).

#### Distributive Justice

One of the major components of organizational justice, distributive justice, centers on employees’ beliefs about compensation fairness when compared to other employees, as well as the propriety of their compensation in terms of task difficulty and responsibilities that are a part of their job (Deutsch, 1985; Folger and Cropanzano, 1998). Distributive justice can also refer to the allocation fairness of other resources such as equipment, work tools, training, and promotional opportunities. Several studies link distributive justice to job satisfaction (McFarlin and Sweeney, 1992; Lowe and Vodanovich, 1995; Cohen-Charash and Spector, 2001; Bakhshi et al., 2009; Ali and Saifullah, 2014).

#### Procedural Justice

Another facet of organizational justice, procedural justice, involves employees’ perceived fairness of the processes and procedures used to make important decisions regarding an organization’s functioning, income distribution, and business operations (Thibaut and Walker, 1975; McFarlin and Sweeney, 1992; Bobocel and Holmvall, 2001). Procedural justice has often been linked with job satisfaction (Moorman et al., 1993; Schappe, 1998; Schmitt and Dörfel, 1999; Cohen-Charash and Spector, 2001; Lambert et al., 2007).

#### Interactional Justice

A third type of organizational justice, interactional justice, is determined by employees’ perceptions of their treatment (e.g., fairness, respect) during exchanges with other individuals, such as other employees, supervisors, upper management, clients, and other representatives of the organization (Bies and Shapiro, 1987; McFarlin and Sweeney, 1992). Much research indicates that employees’ perceptions of interactional justice are positively related to job satisfaction (Cohen-Charash and Spector, 2001; Holmvall and Sidhu, 2007; Ladebo et al., 2008; Ismail and Zakaria, 2009).

#### Status Ambition and Demographic Factors

In addition to organizational attitudes, we examine the potential link between status anxiety and job satisfaction beyond other relevant factors. This includes status ambition, that is, the desire for higher status. This factor tends to show a moderate positive association with status anxiety (Day and Fiske, manuscript in preparation). We also examine our hypothesis by including several background characteristics, some of which have been found to relate to status anxiety (e.g., age, income, perceived socioeconomic status (SES); Day and Fiske, manuscript in preparation), job satisfaction (e.g., gender, age, income, education; Kacmar and Ferris, 1989; Clark and Oswald, 1996; Clark et al., 1996; Pouliakas and Theodossiou, 2003; Kifle, 2013; Batz-Barbarich et al., 2018), or other indicators of well-being (e.g., perceived SES; Singh-Manoux et al., 2003; Schneider, 2019; Rivenbark et al., 2020).

### Overview of Present Research

As outlined, we hypothesize that the experience of status anxiety will negatively relate to workers’ overall job satisfaction. We expect that this relationship will hold even when accounting for several organizational attitudes, including job insecurity and demographic characteristics. To test our ideas, we will conduct a pilot study and a main study. In the pilot study, we will first examine whether status anxiety differs from another organizational attitude, job insecurity, using confirmatory factor analyses. That is, evidence beyond a test of predictive validity (e.g., with each variable explaining unique variance in job satisfaction) would more strongly bolster our assertion that these constructs are separate (Clark and Watson, 1995; Flake et al., 2017; Wang and Eastwick, 2020). After, in our main study, we will test whether status anxiety relates to job satisfaction above and beyond the discussed relevant factors. In both studies, we will examine responses from full-time employed individuals because of the organizational nature of our research questions and to help facilitate uniform responding to a particular job. Learning about the possible unique association between status anxiety and job satisfaction among workers is important, in part, because it will further our understanding of how everyday concerns surrounding social status may link to well-being and professional outcomes. Moreover, studying whether status anxiety relates to job satisfaction may, in turn, help us design better workplace policies targeted at increasing employee satisfaction and well-being.

## Pilot Study

Prior to our central hypothesis test, the goal of the pilot study is to examine whether status anxiety differs from job insecurity, that is, a construct with some potential overlap with status anxiety that relates to job satisfaction. We will rely on a recently developed measure of status anxiety (Day and Fiske, manuscript in preparation), which reflects how this construct has been defined (de Botton, 2004). Although proxy indicators of status anxiety have been used in survey research (Delhey and Dragolov, 2014; Layte and Whelan, 2014; Paskov et al., 2017), the present measure is the first to be conceptually validated across multiple studies (Day and Fiske, manuscript in preparation). However, there are several measures of job insecurity. To strengthen our test of the potential uniqueness of status anxiety, we selected two widely used and well-defined measures of job insecurity for comparison (Oldham et al., 1986; Borg and Elizur, 1992). We sought to examine whether the status anxiety measure would differ from each of these job insecurity measures among full-time workers.

### Materials and Methods

We recruited a sample of American participants for a study on “Lifestyle and Job Attitudes” from Amazon Mechanical Turk (Paolacci et al., 2010; Buhrmester et al., 2011). Crowdsourcing platforms such as Mechanical Turk have been found to facilitate the collection of quality data, including for the study of industrial–organizational topics (see Landers and Behrend, 2015). We also used TurkPrime online software to help with the recruitment process, compensate participants with US $1, and prevent the participation of individuals who completed our prior research studies (Litman et al., 2017). As we were interested in testing our hypothesis on full-time workers, we encouraged accurate responding about employment. Specifically, the study description emphasized that participation (and thus compensation) was possible regardless of employment status (Chandler and Paolacci, 2017). Of the 200 participants, 75% indicated they were employed full-time, 16% part-time, 8% unemployed, and 1% were retired. Only full-time workers (*n* = 150) were directed to complete the pilot study materials, which included a measure of status anxiety and two measures of job insecurity (people that were part-time, retired, or unemployed were directed to complete unrelated tasks, see the Supplementary Material for details). To improve data quality, and as a type of attention check, we excluded three of the participants employed full-time because they failed to provide a meaningful response to either a question about their job title or job responsibilities (leaving these participants in did not change the findings). As all participants indicated that they wished their data to be analyzed, we did not exclude any individuals for this reason. The effective sample consisted of 147 participants (51.0% female, mean_*age*_ = 36.14, SD_*age*_ = 9.71, 78.9% White; 53.3% with a bachelor’s degree or higher).

#### Status Anxiety

Participants completed a five-item measure (α = 0.94) of individuals’ concern and worry over whether their status meets society’s definition of success (Day and Fiske, manuscript in preparation). This measure captures concerns about not being able to move upward, falling to a lower status, as well as feeling too low or stuck in their current position in society, for example, “I worry that my social status will not change.” The measure shows good test–retest reliability (*r* = 0.76) over a 3-week period (Day and Fiske, manuscript in preparation). Participants completed all measures using seven-point rating scales (e.g., 1 = *strongly disagree*, 7 = *strongly agree*), with higher scores indicating higher levels of a construct.

#### Job Insecurity

Participants completed two measures of job insecurity. The Borg and Elizur (1992) job insecurity measure (nine items, α = 0.90) focuses on thoughts and beliefs, as well as feelings of worry and emotional disturbance, associated with the fear of losing one’s job, for example, “The possibility of losing my job puts a lot of strain on me.” The measure of Oldham et al. (1986) (10 items; α = 0.92) is designed to assess individuals’ sense that their future employment with their organization is stable and predictable, for example, “My job is not a secure one.” Both measures provide reasonable assessments of job insecurity (Staufenbiel and König, 2011; Komendat and Didona, 2016).

### Results

To test whether the status anxiety measure differs from the two job insecurity measures, we first examine their association with zero-order correlations. In addition, we conduct separate confirmatory factor analyses to determine whether the status anxiety measure combined with each job insecurity measure is better represented by one or two factors. Evidence of a relatively superior two-factor model would bolster confidence that status anxiety and each job insecurity measure were unique.

Correlations indicated that status anxiety was significantly associated with the job insecurity measures, *r* = 0.373, *p* < 0.001 (Oldham et al., 1986), and *r* = 0.494, *p* < 0.001 (Borg and Elizur, 1992). Both job insecurity measures were also highly correlated, *r* = 0.875, *p* < 0.001. Next, we employed confirmatory factor analyses. Rather than strive for optimal fit, our goal was to compare the relative fit of a one-factor model of status anxiety and job insecurity (i.e., status anxiety and job insecurity are parts of the same concept) to a 2-factor model (i.e., each measure reflects a separate concept), for each job insecurity measure. We used R Studio, lavaan package, to conduct these analyses (see the Supplementary Material), with root mean square error of approximation (RMSEA) closer to 0 and Tucker–Lewis index (TLI) closer to 1, as indicators of better model fit. First, we compared the Day and Fiske (manuscript in preparation) status anxiety measure with the Borg and Elizur job insecurity measure. Results indicated that a two-factor model better fit the data (RMSEA = 0.136, TLI = 0.845) than a single-factor model (RMSEA = 0.245, TLI = 0.498). Similarly, the job insecurity measure of Oldham et al. showed a better fit with a two-factor model (RMSEA = 0.147, TLI = 0.840) than with a single-factor model (RMSEA = 0.251, TLI = 0.531).

### Discussion

Together, these results support the notion that status anxiety and job insecurity are unique constructs. Our confidence in this finding is increased, in part, because status anxiety appears to differ from two independently developed indicators of job insecurity. Testing our main hypothesis in the next study may provide additional evidence of the uniqueness of these constructs. While both job insecurity measures reasonably fit the two-factor models above and appear to be viable indicators of job insecurity, we selected the Borg and Elizur measure to use in our main study, where we examine whether status anxiety will uniquely predict job satisfaction.

## Main Study

### Materials and Methods

We recruited a sample of 401 participants from Amazon’s Mechanical Turk. We used TurkPrime to assist with recruitment and to preclude participation of any individuals from the pilot study and our other past research. Participants signed up for a study on “Lifestyle and Job Attitudes” and were remunerated with US $1. Similar to the pilot study, the study description highlighted that people of “all employment statuses (full-time, part-time, unemployed, etc.)” were eligible and encouraged to participate (Chandler and Paolacci, 2017). We followed the same exclusion procedure as in the pilot study and consistent with our study analysis plan. We initially excluded two participants because they did not want their data analyzed (leaving *n* = 399). Of the remaining, 63.9% (*n* = 255) were employed full-time, 19.5% part-time, 14.5% were unemployed, and 2.0% were retired. As our focus was only on full-time workers, individuals who were part-time employed, unemployed, or retired (*n* = 144) were redirected to another study. To help maintain data quality, we also excluded 14 of the participants employed full-time because they failed to provide a job title or plausible response about their job responsibilities. This left a final effective sample of 241 participants (52.9% female, *mean*_*age*_ = 34.79, *SD*_*age*_ = 9.06, 71.4% White; 50.2% with a bachelor’s degree or higher). To better understand the employment characteristics of the sample, we assessed participants’ job types and industries using the 10-option International Standard Classification of Occupations and the 20-option North American Industry Classification System, respectively. These classifications were selected because of their recognition and frequent use in national and international labor surveys. As seen in Table 1, the most common occupational categories included professionals (29.9%), managers (20.3%), and technicians and associate professionals (14.5%). The most common industries were retail trade (16.2%), professional, scientific, and technical services (14.9%), manufacturing (9.1%), and health care (8.7%). In general, the sample consisted mostly of skilled white-collar workers employed across a wide variety of industries. See the Supplementary Material for complete participant information, study analysis plan, and materials.

**TABLE 1:** Respondents’ professional occupation by the International Standard Classification of Occupations categories, main study.

Job category	Percent
(1) Managers (chief executive officers, senior officials, legislators)	20.3%
(2) Professionals (science, engineering, health, and education)	29.9%
(3) Technicians and associate professionals	14.5%
(4) Clerical support workers	12.4%
(5) Service and sales workers	12.9%
(6) Skilled agricultural, forestry, and fishery workers	0.8%
(7) Craft and related trades workers	3.7%
(8) Plant and machine operators and assemblers	2.5%
(9) Elementary occupations	2.1%
(10) Armed forces	0.8%
Total (*n*)	100% (241)

Participants completed the study online using the Qualtrics survey platform. After indicating demographic characteristics, participants completed the main study measures, including status anxiety; status ambition; job insecurity; occupational self-efficacy; distributive, procedural, and interactional justice; and job satisfaction. The order of measures was counterbalanced such that status anxiety and status ambition were either completed first or the measures of organizational attitudes. All participants completed the job satisfaction measure last. The order of measures did not interact with any of the main variables, and thus, we do not discuss it further. All measures were completed using seven-point rating scales, with higher scores indicating higher levels of the construct.

#### Status Anxiety

As in the pilot study, we used a five-item measure (α = 0.92; Day and Fiske, manuscript in preparation) to assess participants’ concerns and worries about not being able to improve their status, losing status, or being too low in society.

#### Status Ambition

We also assessed desires to increase one’s social and economic standing with a five-item measure (α = 0.88; Day and Fiske, manuscript in preparation). For example, “It is important to take opportunities that can increase your social standing.”

#### Job Insecurity

As in the pilot study, we used the Borg and Elizur (1992) nine-item measure (α = 0.85) to examine thoughts and beliefs, as well as feelings of worry and emotional disturbance, associated with the fear of losing one’s job.

#### Occupational Self-Efficacy

To assess occupational self-efficacy, we used a six-item measure that evaluated participants’ confidence in their job-related skills and overall competence (α = 0.92; Rigotti et al., 2008). For example, “Whatever comes my way in my job, I can usually handle it.”

#### Distributive, Procedural, and Interactional Justice

These measures of fairness and impartiality were based on subcomponents of a broader measure of perceptions of organizational justice (Moorman, 1991). We slightly modified the wording of some items to make the questions answerable for participants employed in different occupations.

#### Distributive Justice

We used a five-item subscale (α = 0.94, Moorman, 1991) to assess beliefs that the financial resources (e.g., salaries, bonuses) were fairly distributed among the employees in their organization. For example, “Please indicate to what extent you are fairly rewarded for the amount of effort you put forth.”

#### Procedural Justice

We used a seven-item subscale (α = 0.94, Moorman, 1991) to evaluate participants’ beliefs that their organizations’ procedures used to make decisions are straightforward and unbiased. For example, “Please indicate how developed the formal procedures in your organization are to have all sides affected by important decisions represented.”

#### Interactional Justice

We used a six-item subscale (α = 0.93, Moorman, 1991) to assess beliefs about how they are treated at work and how much one’s organization’s officials were open in their communication with the employees and receptive of employees’ feedback. For example, “In general, the representatives of this organization consider your viewpoint.”

#### Job Satisfaction

To estimate participants’ overall satisfaction with their jobs, we adopted a five-item version (α = 0.87) of a widely used scale first developed by Brayfield and Rothe (1951), for example, “I feel fairly well satisfied with my present job.” This measure has been adapted for use in numerous studies in organizational psychology, which have validated its reliability and validity (Price, 1997; Judge et al., 2002; Dalal et al., 2012).

#### Demographics

Participants provided a variety of background information including their gender, age, ethnicity, education (eight levels), personal income (12 levels), and perceived SES on a 10-point ladder (Adler et al., 2000).

### Results

Our main goal was to determine whether status anxiety distinctly relates to job satisfaction beyond other relevant factors. We first examine how status anxiety and the other six psychological factors may relate to job satisfaction using zero-order correlations. As our central test, we subsequently conduct a multiple regression to examine which of these main factors uniquely explains variance in participants’ job satisfaction. To examine the robustness of these results, we conduct another multiple regression that additionally controls for various background characteristics.

Zero-order correlations revealed that most of the main study variables were significantly associated with each other (coefficients, as well as means and standard deviations, are displayed in Table 2). The exception was status ambition, which was associated only with status anxiety. Of particular interest was the moderate relationship between status anxiety and lower job satisfaction, *r* = −0.524, *p* < 0.001. Job satisfaction was also negatively related to job insecurity and positively related to occupational self-efficacy, as well as distributive, procedural, and interactional justice.

**TABLE 2:** Means, standard deviations, and correlations among the main study variables.

Variable	Mean	SD	1	2	3	4	5	6	7	8
(1) Status anxiety	3.232	1.517	(–)	0.337***	0.562***	−0.396***	−0.402***	−0.316***	−0.318***	−0.524***
(2) Status ambition	4.584	1.264		(–)	0.059	0.102	0.007	−0.004	0.059	−0.044
(3) Job insecurity	2.909	1.026			(–)	−0.524***	−0.496***	−0.514***	−0.493***	−0.660***
(4) Occupational self-efficacy	5.912	0.892				(–)	0.334***	0.402***	0.409***	0.476***
(5) Distributive justice	4.942	1.399					(–)	0.640***	0.672***	0.596***
(6) Procedural justice	5.110	1.256						(–)	0.802***	0.638***
(7) Interactional justice	5.180	1.303							(–)	0.649***
(8) Job satisfaction	4.996	1.288								(–)

***p < 0.001.

Following our study plan, we conducted a multiple regression to examine whether status anxiety uniquely related to job satisfaction beyond variance explained by status ambition, job insecurity, occupational self-efficacy, and distributive, procedural, and interactional justice (Table 3). Even when job insecurity and the other organizational attitudes were controlled for, status anxiety was still a significant predictor of lower job satisfaction (β = −0.180, *p* = 0.001). Several other variables uniquely related to job satisfaction, including job insecurity (β = −0.278, *p* < 0.001) and interactional justice (β = 0.223, *p* = 0.002). We found associations for procedural justice (β = 0.163, *p* = 0.022) and distributive justice (β = 0.111, *p* = 0.059), with the latter not reaching statistical significance, although the pattern was in the expected direction. Neither status ambition nor occupational self-efficacy significantly predicted satisfaction with one’s job (β*s* < 0.065, *ps* > 0.199). Together, these variables explained approximately 60% of the variance in job satisfaction (Adjusted *R2* = 0.611).

**TABLE 3:** Psychological predictors of job satisfaction, main study.

	*b*	SE	*b* 95% CI	β	*t*	*p*
Status anxiety	−0.152	0.047	[−0.244, −0.061]	−0.180	−3.273	0.001
Status ambition	0.013	0.046	[−0.077, 0.104]	0.013	0.290	0.772
Job insecurity	−0.349	0.072	[−0.494, −0.207]	−0.278	−4.853	<0.001
Occupational self-efficacy	0.093	0.072	[−0.050, 0.235]	0.064	1.285	0.200
Distributive justice	0.102	0.054	[−0.004, 0.208]	0.111	1.900	0.059
Procedural justice	0.167	0.073	[0.024, 0.311]	0.163	2.303	0.022
Interactional justice	0.220	0.072	[0.078, 0.362]	0.223	3.059	0.002
*R*^2^ (adjusted *R*^2^)					0.622	(0.611)
Constant					3.393	<0.001

To further test the ability of status anxiety to explain job satisfaction, we also explored the possible roles of relevant demographic factors. For example, an examination of the zero-order correlations revealed that both personal income (*r* = 0.196, *p* = 0.002) and perceived SES (*r* = 0.210, *p* = 0.001) were associated with job satisfaction. Thus, we conducted another multiple regression, controlling for a variety of background variables (see the Supplementary Material for details on all exploratory analyses). Specifically, on the first step, we entered gender, age, education, personal income, and perceived SES, and on the second step, we entered the same seven psychological predictors as above. Although these demographic variables initially accounted for approximately 5% of the job satisfaction variance on step 1 (adjusted *R*^2^ = 0.046), for example, perceived SES (β = 0.159, *p* = 0.043), none of these individual factors were significant predictors on Step 2 when the main study variables were included. Importantly, status anxiety remained a significant predictor of job satisfaction (β = −0.175, *p* = 0.002), as did job insecurity and interactional justice. Procedural and distributive justice showed positive associations with job satisfaction just under or approaching the criterion for significance (*p* = 0.020, *p* = 0.054, respectively).

Other exploratory analyses provided additional support for the main findings. Although we excluded some participants based on our study analysis plan, we note that including these participants does not meaningfully change the main study findings (e.g., status anxiety still significantly predicts job satisfaction). We also did not find evidence that suggests multicollinearity is a strong concern among the predictor variables, despite the tendency for some factors to moderately or strongly correlate.

### Discussion

Our main study supported the hypothesis that status anxiety uniquely relates to job satisfaction. Critically, this relationship remained when controlling for several organizational attitudes, such as job insecurity, and a variety of individual characteristics, such as income and perceived socioeconomic status. Consistent with prior research, other organizational variables were also associated with job satisfaction (De Witte, 1999; Cohen-Charash and Spector, 2001; Holmvall and Sidhu, 2007; Lambert et al., 2007). Specifically, job insecurity was related to lower job satisfaction, whereas interactional justice and procedural justice were related to higher satisfaction. Several variables did not show such associations. Status ambition showed little to no relation with job satisfaction or with other organizational attitudes. Occupational self-efficacy correlated with job satisfaction, but showed a weak to non-existent association when controlling for other variables. Distributive justice also showed a relatively weak association with job satisfaction. These latter findings contrast with other research (Bakhshi et al., 2009; Guarnaccia et al., 2018). One possibility may be that other variables we included (e.g., status anxiety, job insecurity) may better explain the initially detected associations between occupational self-efficacy, distributive justice, and job satisfaction. Alternatively, these organizational variables may have more complex relationships with job satisfaction, depend on individual differences, or operate under certain conditions (Holmvall and Sidhu, 2007; Borgogni et al., 2010; Bobocel and Gosse, 2015; Maggiori et al., 2016). These possibilities could be confirmed in future research.

## General Discussion

It is not uncommon to be concerned, uncertain, or worried about whether one may be successful in life. In particular, status anxiety captures people’s broad concerns about not achieving a higher status, losing status, or being stuck at a particular rank in society (de Botton, 2004). The present research examined whether the experience of status anxiety among full-time workers differs from other well-researched psychological phenomena and sheds light on a consequential indicator of well-being: satisfaction with one’s job. In a pilot study, we found that status anxiety did not overlap with feelings of job insecurity. Moreover, our main study identified a unique relationship between higher status anxiety and lower job satisfaction beyond what was accounted for by several other known predictors of job satisfaction, including job insecurity and background characteristics. In other words, concerns about meeting societal standards of success explain, in part, why workers can be dissatisfied with their jobs. Although the assertion that status anxiety can affect health and well-being outcomes has been around for a decade (Wilkinson and Pickett, 2009), this research is the first to empirically document the unique relationship between status anxiety and an indicator of employee well-being in organizational settings.

Consistent with past research, other organizational factors played a role in job satisfaction. For example, we found that feeling secure about one’s job and perceiving to be treated well by one’s organization (e.g., respectfully, truthfully) helped explain higher job satisfaction (De Witte, 1999; Ismail and Zakaria, 2009). Distributive and procedural justice beliefs about one’s organization also positively associated with job satisfaction (McFarlin and Sweeney, 1992; Cohen-Charash and Spector, 2001). However, not all examined factors uniquely related to job satisfaction; in particular, we note the lack of association between status ambition and job satisfaction. Although status ambition is often associated with status anxiety (Day and Fiske, manuscript in preparation), it appears the goal of seeking higher status in society may not impede or elevate personal satisfaction with one’s job, at least on its own or in a straightforward manner. Moreover, a variety of participants’ background characteristics did not explain workers’ degree of job satisfaction. For example, although perceived socioeconomic status has been linked with multiple well-being outcomes (e.g., Singh-Manoux et al., 2003; Rivenbark et al., 2020) and was related to job satisfaction in this research, this association effectively disappeared when accounting for other psychological variables. This suggests that psychological factors that capture individual experiences with evaluative or affective components may be better suited for explaining well-being related outcomes (e.g., Smith and Pettigrew, 2014; Jiang and Lavaysse, 2018) than factors focused only on perceptions of objective conditions (e.g., subjective rank; Adler et al., 2000). This possibility, and whether it is limited to organizational settings, could be examined in future research.

### Limitations

There are some limitations to the present studies. For instance, because our research was correlational, it is not possible to determine the causality of the relationship between status anxiety and job satisfaction or among the other examined variables. Status anxiety involves broad concerns about one’s standing in society that may arise in many contexts, including those outside of the workplace environment (de Botton, 2004; Wilkinson and Pickett, 2009). Researchers have also argued that the experience of status anxiety over time likely has downstream consequences on individuals’ overall well-being (Wilkinson and Pickett, 2009; Buttrick et al., 2017). While the novel findings of the present research are consistent with this possibility, additional research would be needed, such as with longitudinal or experimental designs, to determine the causal nature of the association between status anxiety and job satisfaction (e.g., see De Witte et al., 2016).

Although this research provided an initial demonstration of the link between status anxiety and job satisfaction, questions remain. Status anxiety is believed to affect well-being and has been well-defined as a concept, but there lacks a strong theory to provide insight into underlying processes and boundary conditions. In the introduction, we speculated about several processes in which status anxiety may affect job satisfaction (e.g., through lack of workplace fulfillment, strain on relationships) and some conditions that may exacerbate it (e.g., lack of advancement). Testing such possibilities is beyond the scope and goals of this early-stage research. However, once additional evidence accumulates, future research could strengthen this area by theorizing how status anxiety may associate with job satisfaction and under what conditions this association may be stronger or weaker (e.g., moderators and individual differences). In turn, this may provide greater insight into a testable research agenda.

Our research samples consisted of full-time workers in a variety of different occupations and industries. Many were also white-collar workers and well-educated. As the experience of status anxiety should not be limited to a particular profession, industry, or status level (e.g., Gill, 2015; Day and Fiske, manuscript in preparation), this occupation and industry diversity allowed for broad tests of whether status anxiety differed from other constructs and related to job satisfaction. In our main study, we found robust relationships between status anxiety, job insecurity, interactional justice, and job satisfaction and explained a reasonable amount of the total variance in respondents’ job satisfaction. However, some of these factors, such as job insecurity, may be more strongly experienced among other occupational and social class groups (e.g., Erlinghagen, 2008). Thus, confidence in our findings may be bolstered through recruitment of even larger and more diverse samples, including some that are from other cultures and are more representative of the upper-, middle-, and working-class populations.

In addition to testing our hypothesis, this research helped further validate a measure of status anxiety (Day and Fiske, manuscript in preparation), for example, by revealing how status anxiety is associated with conceptually related constructs, but is also distinct. Although we employed widely used measures of other psychological constructs, including two separate measures of job insecurity in our pilot study, we acknowledge that research assessments are evolving, and other validated measures exist (e.g., Shoss, 2017). Thus, additional support for the present findings may be gained from the use of new and varied measurement techniques.

### Implications

The results of the present studies have potential significance for the future of social and organizational research. Job satisfaction is a major determinant of quality of life and, to some degree, can affect employee productivity, performance, and commitment to organizations (Dailey and Kirk, 1992; Moorman, 1993; Judge et al., 2001; Allan et al., 2018). Our finding that status anxiety is directly related to job satisfaction potentially opens up new research questions about the association between status anxiety and other organizational outcomes and situations. For example, future research could examine whether those who experience higher status anxiety show less organizational commitment, higher turnover intentions, or less employee efficiency. It may also be fruitful to consider whether workers’ experience of status anxiety can provide insight into status-related workplace behaviors or have conditional effects on related experiences such as job insecurity (Shoss, 2017). There may also be antecedents of status anxiety that are unique to workplaces. For instance, as economic inequality may heighten status anxiety, which then may negatively affect well-being in the population (Wilkinson and Pickett, 2009), future research could also examine the possible mediating role of status anxiety in reactions to pay inequalities in the workplace, perhaps especially among those lower in status (e.g., Card et al., 2012). Similarly, as economic crises are consistently linked to worse psychological well-being (Van Hal, 2015; Martin-Carrasco et al., 2016; Marazziti et al., 2020), it may be worthwhile to explore how the relationship between status anxiety and lower job satisfaction may be affected by such societal events.

The link between the experience of status anxiety among workers and job dissatisfaction may also have implications for workplace programs and policies. For example, additional research may uncover methods to reduce the experience of status anxiety. This could potentially increase job satisfaction and, in turn, employee performance and productivity (Judge et al., 2001). One possibility may be through adopting an organizational ideology of gratitude that promotes this quality among employees (DeSteno, 2018) and emphasizes how each individual employee brings value to the organization, regardless of their status. Or more broadly, through organizational restructuring (e.g., by minimizing existing pay gaps), which may reduce perceived status differences among higher occupational levels and thus perhaps also the significance of status-related concerns. Although lofty, there are examples of organizations significantly altering their pay structures (e.g., Cohen, 2015). Another possibility, consistent with the link between job satisfaction and career development needs (Chen et al., 2004), would be to support employees’ mobility within organizations through frequent career development conversations as well as encouragement to try on new roles and develop skills that may make them better suited for their next career move. Encouraging employees to be proactive with their careers can create an atmosphere of support between organizations and employees and may also ameliorate some anxiety and uncertainty employees might experience about the future of their social status. The efficaciousness of the assumed outcomes of such policy changes could be examined in future research.

## Conclusion

Many individuals are concerned about whether they will move up in life or be stuck in their position in society. Many also spend a considerable amount of their life at work. Their degree of satisfaction with their jobs is consequential not only for their organization, but also for their overall well-being. Our research was the first to document that broad concerns about societal status, that is, status anxiety, can help explain job dissatisfaction. This association was unique and could not be readily explained by other prominent organizational factors known to predict job satisfaction. Thus, this research also provides novel psychological insight into our understanding of the concept of status anxiety. Finally, it also opens a number of opportunities for future inquiries within organizational psychology into people’s status-related concerns, their well-being, job attitudes, and potential impacts on workplace outcomes.

## Data Availability Statement

The datasets generated for this study are available on request to the corresponding author.

## Ethics Statement

The studies involving human participants were reviewed and approved by the Interdisciplinary Committee on Ethics in Human Research, Memorial University of Newfoundland. The participants provided informed consent to participate in this research.

## Author Contributions

AK and MD designed the study, analyzed the data, and wrote the manuscript. AK collected the data. Both authors contributed to the article and approved the submitted version.

## Conflict of Interest

The authors declare that the research was conducted in the absence of any commercial or financial relationships that could be construed as a potential conflict of interest.
