# Supplementary material for: Concerned Whether You’ll Make It in Life? Status Anxiety Uniquely Explains Job Satisfaction
Source: Front Psychol. 2020 Jul 10;11:1523. doi: 10.3389/fpsyg.2020.01523 (PMC7381271; doi:10.3389/fpsyg.2020.01523)
Supplement: Supplementary file 1 [file Data_Sheet_1.pdf]

Supplemental File for

Keshabyan, A. & Day, M. V. (2020). Concerned whether you'll make it in life? Status anxiety uniquely explains job satisfaction. *Frontiers in Psychology*.

Table of Contents

**Pilot Study**	
-Participants	2
-Procedure and Materials	3
-Results	4
-Confirmatory Factor Analyses	4
**Main Study**	6
-Participants	6
-Study Analysis Plan	6
-Materials	6
-Predictor Variables	6
-Dependent Variable	8
-Additional Analyses	8
-Correlations Among Main Study and Background Variables	9
-Psychological and Background Predictors of Job Satisfaction	10
-Psychological Predictors of Job Satisfaction with all Participants	10
-Exploration of Multicollinearity	11
**References**	11

Pilot Study

Participants

The following are some additional details about the effective sample of 147 full-time workers. Participants ethnicity: White (78.9%); Black (10.9%), Asian (4.1%), Hispanic (4.1%), Other (2.0%). Education: High School graduate or equivalent (10.9%), Some college (19.0%), Associate degree (17.0%), Bachelor's degree (39.5%), Master's degree (11.6%), Doctorate or Professional degree (2.0%). Median household income: \$60,001-\$80,000. Perceived SES: *M* = 4.99, *SD* = 1.58. All participants were U.S. residents. The sample was somewhat heterogeneous in terms of occupations and industries (see Tables S1 and S2). Similar to the Main Study, the most common occupational categories included Professionals (30.6%), Clerical Support Workers (17.7%) and Managers (18.4%). Common industries included: Retail Trade (12.2%), Professional, Scientific, and Technical Services (11.6%), and Health Care (8.8%).

Table S1. *Respondents' Professional Occupation by International Standard Classification of Occupations*

	Pilot Study	Main Study
Job Category	Percent	Percent
1. Managers (CEOs, senior officials, legislators)	18.4%	20.3%
2. Professionals (science, engineering, health, and education)	30.6%	29.9%
3. Technicians and Associate Professionals	11.6%	14.5%
4. Clerical Support Workers	17.7%	12.4%
5. Service and Sales Workers	15.6%	12.9%
6. Skilled Agricultural, Forestry, and Fishery Workers	0.7%	0.8%
7. Craft and Related Trades Workers	2.0%	3.7%
8. Plant and Machine Operators and Assemblers	2.0%	2.5%
9. Elementary Occupations	1.4%	2.1%
10. Armed Forces	0%	0.8%
Total (N)	100% (147)	100% (241)

Table S2. *Proportion of Respondents Employed in Job Industries Using the North American Industry Classification System*

	Pilot Study	Main Study
Industry	Percent	Percent
1. Agriculture, forestry, fishing, and hunting	1.4%	1.2%
2. Utilities (e.g. electric, gas, water)	0.7%	1.2%
3. Construction	1.4%	4.6%
4. Manufacturing	3.4%	9.1%
5. Wholesale trade	4.1%	1.7%
6. Retail trade	12.2%	16.2%
7. Transportation and warehousing (e.g. air, water, ground, pipelines)	3.4%	2.5%
8. Information and cultural industries (publishing, broadcasting, and telecommunications)	3.4%	6.2%
9. Real estate and rental leasing	2.7%	1.2%
10. Professional, scientific, and technical services	11.6%	14.9%

11. Management of companies and enterprises	6.1%	1.2%
12.Administrative and support, waste management and remediation services	4.8%	3.3%
13. Educational services	5.4%	7.1%
14. Healthcare	8.8%	8.7%
15. Social assistance	2.7%	0.8%
16. Arts, entertainment, and recreation	5.4%	5.4%
17. Accommodation and food services	6.1%	2.5%
18. Public administration	5.4%	2.5%
19. Other	10.9%	9.1%
Total (N)	100% (147)	100% (241)

Procedure and Materials

To encourage truthful responding to the employment question, the study advertisement listed that "All employment statuses (full-time, part-time, unemployed, etc) are eligible to participate" in the study. Participants completed the study online using the Qualtrics platform and were compensated with US $1.00. After providing background characteristics, including their employment status, participants completed the main study measures, including status anxiety and two measures of job insecurity (see below). The order of measures was counterbalanced in such a way that half of the participants completed the status anxiety scales before job insecurity scales; while the other half completed the job insecurity scales first and then status anxiety scale. Employed participants were asked two open-ended questions about their job, which were part of our exclusion criteria, as in the main study. This included their job title, "What is your job title?" and a brief description of their job "If you had to briefly describe your job to a new friend/acquaintance, how would you describe your most essential duties and day-to-day activities?" For exploratory purposes, participants also completed a question about their political orientation, a 3-item measure of powerlessness, and a 5-item measure of status ambition. Those that indicated they were not full-time workers were re-directed to complete other research after completing the employment and background questions.

[Status Anxiety – Day & Fiske]

Instructions: Please indicate how much you agree or disagree with the following statements.
(1 = *strongly disagree*, 7 = *strongly agree*)
1. I feel anxious that I will be stuck in my position for life.
2. I am very concerned that I won't be able to achieve my career goals.
3. I worry that I might become lower in social standing.
4. I am concerned that my current position in life is too low.
5. I worry that my social status will not change.

[Job Insecurity – Borg & Elizur]

Instructions: Please, indicate how much you agree with the following statements about your job.
(1 = *strongly disagree*, 7 = *strongly agree*)
1. I am concerned about the possibility of being dismissed.
2. The possibility of losing my job puts a lot of strain on me.
3. I would feel stressed if I had to fight for my job.
4. I believe that my job is secure.

5. I believe that my career is secure.
6. I clearly know my chances for advancement in the coming years.
7. In my opinion, I will keep my job in the near future.
8. In my opinion, I will be employed for a long time in my present job.
9. I look forward with confidence to the introduction of new technologies.
Reverse-scored items: 4, 5, 6, 7, 8, 9

[Job Insecurity – Oldham et al]
Instructions: Please indicate how accurately the following statements represent your job situation. (1 = *very inaccurate*, 7 = *very accurate*)
1. I will be able to keep my present job as long as I wish.
2. My organization will not cut back on the number of hours I work each week.
3. If my current organization were facing economic problems, my job would be the first to go.
4. I am confident that I will be able to work for my organization as long as I wish.
5. My job will be there as long as I want it.
6. If my job were eliminated, I would be offered another job in my current organization.
7. Regardless of economic conditions, I will have a job at my current organization.
8. I am secure in my job.
9. My current organization would transfer me to another job if I were laid off from my present job.
10. My job is not a secure one.
Reverse-scored items: 1, 2, 4, 5, 6, 7, 8, 9

Results

Confirmatory Factor Analysis

To determine the number of factors we primarily relied on prior research, however we also took eigenvalues for the models into consideration (e.g., Browne, 2001). Eigenvalue, in the context of factor analysis, represents the variance in all the variables associated with a specific factor. We used the Root Mean Square Error of Approximation (RMSEA) and Tucker-Lewis Index (TLI) to evaluate the overall model fit. The RMSEA is an absolute fit index which assesses how far the explored model is from a perfect fit, with smaller RMSEA values indicating a better model fit (Xia & Young, 2018; Jöreskog & Sörbom, 1993). The TLI compares the fit of the hypothesized model with that of a baseline model (worst-fit model) and the greater TLI stands for a relatively better model fit (Xia & Young, 2018; Bentler & Bonett, 1980). As only 4% of our data contained missing values and the data was implied to be missing completely at random, listwise exclusion was used to deal with the missing values (Kang, 2013).

Two-factor analyses: See Table S3 for the factor loadings of the CFA comparing status anxiety and the Borg & Elizur (1992) job insecurity measure, and Table S4 for the factor loadings for status anxiety and the Oldham et al. (1986) job insecurity measure, as described in the manuscript.

In exploratory analyses we also examined whether 3-factor models would provide better model fits. For example, for the Oldham measure, there appeared to be a potential third factor with a 1.45 Eigenvalue (i.e., above the typical cut-off of 1). Exploratory factor analysis also revealed that two items from this measure (#6 & #9) may fit on a third factor (separate from the status anxiety and other job insecurity items). Post-hoc, these items appear to represent perceptions of one's lateral employability. However, the CFA metrics for this 3-factor model

show only a modest improvement (RMSEA = 0.100, TLI = 0.925), and do not appear to corroborate the single-factor design of the Oldham measure. Similarly, we also explored a 3-factor model involving the Borg and Elizur measure. Although the Eigenvalue for a third factor was low (0.95), we examined a three-factor model with status anxiety as one factor, and the cognitive and affective job insecurity items separated into two factors (which has some conceptual grounding to be separate). However, there was only modest improvement of model fit (RMSEA = 0.123, TLI = 0.873). Together, there is not much combined theoretical and statistical evidence justifying these three-factor models, and the evidence also does not appear to challenge the viability of status anxiety being a separate factor from either measure of job insecurity.

Table S3: *Factor loadings of Status Anxiety and Job Insecurity (Borg & Elizur, 1992)*

	Factor	
Item	1	2
JI_1	.843	
JI_2	.797	
JI_3	.294	
JI_4	.869	
JI_5	.805	
JI_6	.653	
JI_7	.761	
JI_8	.810	
JI_9	.594	
SAx_1		.879
SAx_2		.878
SAx_3		.882
SAx_4		.835
SAx_5		.876

Table S4: *Factor loadings of Status Anxiety and Job Insecurity (Oldham et al., 1986)*

	Factor	
Item	1	2
JI_1	.899	
JI_2	.620	
JI_3	.558	
JI_4	.936	
JI_5	.956	
JI_6	.427	
JI_7	.832	

JI_8	.902	
JI_9	.434	
JI_10	.803	
SAx_1		.880
SAx_2		.877
SAx_3		.882
SAx_4		.836
SAx_5		.875

Main Study

Participants

The effective sample of 241 full-time workers had the following additional characteristics: White (71.4%); Black (11.6%), Asian (7.9%), Hispanic (5.0%), Native American (1.7%), Middle Eastern (0.4%), Other (1.7%) and 0.4% undisclosed. Education: Some High School (0.4%), High School graduate or equivalent (10.0%), Some college (25.3%), Associate degree (14.1%), Bachelor's degree (39.0%), Master's degree (10.0%), Doctorate or Professional degree (1.2%). Median personal income: $30,001-$45,000. Median household income: $60,001-$80,000. Perceived SES: $M = 4.88$, $SD = 1.66$. Residence: 99.6% indicated they U.S. residents, with 0.4% undisclosed. See Tables S1 and S2 above for complete information on participants' occupations and employment industries.

Study Analysis Plan

Below is a link to our online document that details our study plan and analysis. This includes information on our plan to recruit 400 participants, exclusion criteria, hypotheses, key study measures, and planned analysis to test our hypotheses. This document is for review purposes and thus does not disclose author information.
https://aspredicted.org/blind.php?x=rp2m7h

We used the AsPredicted platform to document our study plan. At the time, we were new to preregistration. Although we decided on the vast majority of these details prior to data collection, we did not officially preregister them until one day after data collection was complete. Thus, we have opted to describe this information as our study analysis plan, and do not claim it is an official preregistration. Nonetheless, we have followed the plan closely, for example, in terms of recruitment numbers, exclusion, and describing additional analyses as exploratory.

Study Materials

Predictor Variables

[Status Anxiety & Job Insecurity]

Participants completed the same measures of status anxiety and job insecurity (Borg & Elizur) as listed in the pilot study above.

[Status Ambition]

Instructions: Please indicate how much you agree or disagree with the following statements.

(1 = *strongly disagree*, 7 = *strongly agree*)
1. It is important to take opportunities that can increase your social standing.
2. Overall, I would be better off if I had a position that was higher in rank.
3. Raising my social status is an important life goal.
4. I would prefer to be at least one position higher than my current position.
5. My future goals include increasing my position in life.

[Occupational Self-efficacy]
Instructions: Please indicate how true the following statements are true of you.
(1 = *not at all true,* 7 = *completely true*)
1. I can remain calm when facing difficulties in my job because I can rely on my abilities.
2. When I am confronted with a problem at my job, I can usually find several solutions.
3. Whatever comes my way in my job, I can usually handle it.
4. My past experiences in my job have prepared me well for my occupational future.
5. I meet the goals that I set for myself in my job.
6. I feel prepared for most of the demands in my job.

[Distributive Justice]
Instructions: We are interested in your opinions about the fairness of procedures in the organization you work for.
Please, indicate to what extent you are fairly rewarded… (1 = *very unfairly*, 7 = *very fairly*)
1. ...considering the responsibilities that you have
2. ...in view of the amount of experience that you have had
3. ...for the amount of effort that you put forth
4. ...for the work that you have done well
5. ...for the stresses and strains of your job

[Procedural Justice]
Instructions: Please indicate how developed the formal procedures in your organization are to...
(1 = *not developed*, 7 = *fully developed*)
1. ...collect accurate information necessary for making important decisions
2. ...provide opportunities to appeal or challenge decisions
3. ...have all sides affected by important decisions represented
4. ...generate standards so that decisions could be made with consistency
5. ...hear the concerns of all those affected by important decisions
6. ...provide useful feedback regarding decisions and their implementation
7. ...allow for requests for clarification and additional information about important decisions

[Interactional Justice]
Instructions: In general, representatives of this organization… (1 = *strongly disagree,* 7 = *strongly agree*)
1. ...consider your viewpoint
2. ...are able to suppress personal biases
3. ...provide you with timely feedback about important decisions and their implication
4. ...treat you with kindness and consideration
5. ...show concern for your rights as an employee

6. ...take steps to deal with you in a truthful manner

Dependent Variable

[Job Satisfaction]
Instructions: Please, indicate how much you agree or disagree with the following statements about your job. (1 = *strongly disagree*, 7 = *strongly agree*)
1. I feel fairly well satisfied with my present job.
2. Most days I am enthusiastic about my work.
3. Each day of work seems like it will never end.
4. I find real enjoyment in my work.
5. I consider my job rather unpleasant.
Reverse-scored items: 3, 5

For exploratory purposes, participants also completed a question about their political orientation.

Additional Analyses
(See below)

Table S5: *Correlations Among Main Study and Background Variables.*

Variable	1	2	3	4	5	6	7	8	9	10	11	12	13
1. Status Anxiety	(-)	.34***	.56***	-.40***	-.40***	-.32***	-.32***	-.52***	-.03	-.10	.02	-.17**	-.15*
2. Status Ambition		(-)	.06	.10	.01	< -.01	.06	-.04	.06	-.10	.09	.05	.07
3. Job Insecurity			(-)	-.52***	-.50***	-.51***	-.49***	-.66***	< .01	< -.01	.04	-.18**	-.18**
4. Occupational Self-efficacy				(-)	.33***	.40***	.41***	.48***	-.05	.19***	-.03	.11	.12
5. Distributive Justice					(-)	.64***	.67***	.60***	< -.01	-.02	< -.01	.33***	.24***
6. Procedural Justice						(-)	.80***	.64***	< .01	< -.01	-.06	.23***	.16*
7. Interactional Justice							(-)	.65***	.08	-.04	-.06	.21**	.21**
8. Job Satisfaction								(-)	< -.01	.07	-.02	.20**	.21**
9. Gender									(-)	-.14*	.02	.15*	.10
10. Age										(-)	< -.01	.09	.01
11. Education											(-)	.32***	.24***
12. Income												(-)	.58***
13. Perceived SES													(-)

Note. *** $p < .001$,** $p < .01$, * $p < .05$; Gender (0 = Female, 1 = Male)

Table S6: *Psychological and Background Predictors of Job Satisfaction*

Step 1	*b*	SE	*b* 95% CI	ß	*t*	*p*
Gender	-.089	.167	[-.419, .241]	-.034	-.531	.596
Age	.006	.009	[-.012, .025]	.045	.702	.483
Education	-.105	.069	[-.241, .031]	-.103	-1.527	.128
Income	.096	.057	[-.016, .208]	.137	1.689	.093
Perceived SES	.123	.060	[.004, .242]	.159	2.039	.043
R^2 (Adjusted R^2)					.066	(.046)
Constant					4.248	< .001
Step 2						
Gender	-.038	.109	[-.254, .177]	-.015	-.351	.726
Age	.008	.006	[-.004, .020]	.057	1.312	.191
Education	.025	.045	[-.063, .114]	.025	.563	.574
Income	-.038	.038	[-.113, .038]	-.054	-.984	.326
Perceived SES	.035	.039	[-.043, .113]	.045	.889	.375
Status Anxiety	-.149	.048	[-.242, -.055]	-.175	-3.131	.002
Status Ambition	.016	.047	[-.077, .109]	.016	.343	.732
Job Insecurity	-.355	.073	[-.498, -.211]	-.282	-4.863	< .001
Occupational Self-efficacy	.070	.076	[-.079, .219]	.049	.931	.353
Distributive Justice	.108	.056	[-.002, .219]	.118	1.935	.054
Procedural Justice	.173	.074	[.027, .318]	.169	2.335	.020
Interactional Justice	.224	.074	[.078, .370]	.227	3.018	.003
R^2 (Adjusted R^2)					.627	(.607)
Constant					3.072	< .001

Note: Gender (0 = Female, 1 = Male)

Table S7: *Psychological Predictors of Job Satisfaction with all Participants*

	b	SE	*b* 95% CI	ß	*t*	*p*
Status Anxiety	-.143	.045	[-.231, -.055]	-.170	-3.204	.002
Status Ambition	.020	.046	[-.070, .110]	.019	.433	.665
Job Insecurity	-.378	.071	[-.519, -.238]	-.291	-5.311	< .001
Occupational Self-efficacy	.081	.070	[-.057, .219]	.056	1.154	.250
Distributive Justice	.107	.054	[.002, .213]	.115	2.002	.046
Procedural Justice	.124	.071	[-.016, .264]	.118	1.738	.083
Interactional Justice	.266	.070	[.129, .403]	.270	3.833	< .001
R^2 (Adjusted R^2)					.626	(.615)
Constant					3.416	< .001

Note: This exploratory analysis includes participants that were excluded from all other analyses because they violated the study inclusion criteria.

Exploration of Multicollinearity

As evident in the main variable correlations, many of the predictor variables associated with each other. Thus, we examined multicollinearity statistics to see whether there was cause for particular concern for interpreting the regression results. There are various guidelines for interpreting multicollinearity, however, a Variance Inflation Factor (VIF) in excess of 5 may be problematic. Although two of the factors from established justice scales had relatively higher VIFs (procedural and interactional justice), none of the VIFs for the predictor variables show reason for any strong concern about multicollinearity. Such concerns may also be partly reduced as a majority of the variance in job satisfaction was explained by the main predictors, which themselves were measured with high reliability (e.g., Grewal, Cote, & Baumgartner, 2004).

Table S8: *Multicollinearity Coefficients for Job Satisfaction*

	VIF
Status Anxiety	1.856
Status Ambition	1.250
Job Insecurity	2.020
Occupational Self-efficacy	1.545
Distributive Justice	2.100
Procedural Justice	3.097
Interactional Justice	3.269

References

Bentler, P.M., & Bonett, D.G. (1980). Significance tests and goodness of fit in the analysis of covariance structures. *Psychological Bulletin, 88*(3), 588-606.

Browne, W.M. (2001). The latent structure of memory: A confirmatory-factor analytic study of memory distinctions. *Multivariate Behavioral Research, 36*(1), 29-51.

Grewal, R., Cote, J. A., & Baumgartner, H. (2004). Multicollinearity and measurement error in structural equation models: Implications for theory testing. *Marketing Science, 23*(4), 519-529.

Jöreskog, K. G., & Sörbom, D. (1993). LISREL 8: Structural equation modeling with the SIMPLIS command language. Chicago, IL, US: Scientific Software International; Hillsdale, NJ, US: Lawrence Erlbaum Associates, Inc.

Kang, H. (2013). The prevention and handling of the missing data. *Korean Journal of Anesthesiology, 64*(5), 402-406.

Xia, Y. & Young, Y. (2018). RMSEA, CFI, and TLI in structural equation modelling with ordered categorical data: The story they tell depends on the estimation methods. *Behavior Research Methods, (1999)*, 1-20.
